# Introductory notions on atypical Alzheimer's disease

**DOI:** 10.1002/alz70857_102188

**Published:** 2025-12-25

**Authors:** Neus Falgàs, Nick Corriveau‐Lecavalier

**Affiliations:** ^1^ Unitat d'Alzheimer i Altres Trastorns Cognitius, Servei de Neurologia, Hospital Clínic, Barcelona, Spain; ^2^ Department of Neurology, Mayo Clinic, Rochester, MN, USA

## Abstract

This presentation will first provide an overview of the rationale behind this Perspective Session bringing the Atypical Alzheimer's disease (AD) and Clinical Trials ISTAART Professional Interest Area (PIAs) together. We will then highlight foundational notions about atypical forms of Alzheimer's disease (AD) and critical shortcomings in current clinical trial designs that fail to address the unique needs of these populations. We will first briefly describe the distinct clinical and biological features of atypical AD variants, including posterior cortical atrophy (PCA), logopenic variant primary progressive aphasia (lvPPA), dysexecutive AD (dAD), and behavioral AD (bvAD). These variants are typically characterized by early‐onset symptoms and predominant non‐memory impairments, factors that have contributed to the significant underrepresentation of these patients in large‐scale clinical trials. Special attention will be given to recent FDA‐approved amyloid‐targeting therapies, which have largely focused on the “typical” amnestic, late‐onset presentation of AD, further underscoring the urgent need for inclusive research efforts for the broader phenotypic spectrum of the disease. We will identify critical gaps in clinical trial design for atypical AD and propose early strategic avenues to address these challenges. Key considerations include optimizing enrollment strategies, developing syndrome‐specific biological and clinical endpoints, and fostering advocacy to promote accurate diagnosis, equitable representation, and improved outcomes for these underrepresented populations.

